# Supplementary material for: Modeling glioblastoma heterogeneity as a dynamic network of cell states
Source: Mol Syst Biol. 2021 Sep 16;17(9):e10105. doi: 10.15252/msb.202010105 (PMC8444284; doi:10.15252/msb.202010105)
Supplement: Supplementary file 6 — Source Data for Figure 5 [file MSB-17-e10105-s004.zip › Figure5A_sourcedata/GSEA_3017/nosigdb_stateF.GseaPreranked.1622795015644/RICHARDS_NOWAKOWSKI_SCIENCE_2017_IPC_DIV1_UPREG.html]

Details for gene set RICHARDS\_NOWAKOWSKI\_SCIENCE\_2017\_IPC\_DIV1\_UPREG[GSEA]

|  || Dataset | state33017 |
| Phenotype | NoPhenotypeAvailable |
| Upregulated in class | na\_neg |
| GeneSet | RICHARDS\_NOWAKOWSKI\_SCIENCE\_2017\_IPC\_DIV1\_UPREG |
| Enrichment Score (ES) | -0.7304098 |
| Normalized Enrichment Score (NES) | -5.814574 |
| Nominal p-value | 0.0 |
| FDR q-value | 0.0 |
| FWER p-Value | 0.0 |
Table: GSEA Results Summary

  

Fig 1: Enrichment plot: RICHARDS\_NOWAKOWSKI\_SCIENCE\_2017\_IPC\_DIV1\_UPREG      
 Profile of the Running ES Score & Positions of GeneSet Members on the Rank Ordered List

  

| PROBE | GENE SYMBOL | GENE\_TITLE | RANK IN GENE LIST | RANK METRIC SCORE | RUNNING ES | CORE ENRICHMENT || 1 | MDM2 |  |  | 12 | 1.421 | 0.0005 | No |
| 2 | SESN3 |  |  | 56 | 0.908 | -0.0177 | No |
| 3 | DAAM1 |  |  | 175 | 0.618 | -0.0763 | No |
| 4 | KAT2B |  |  | 193 | 0.594 | -0.0823 | No |
| 5 | TRIAP1 |  |  | 208 | 0.584 | -0.0869 | No |
| 6 | SRGAP2C |  |  | 238 | 0.563 | -0.0993 | No |
| 7 | PIK3R3 |  |  | 251 | 0.553 | -0.1030 | No |
| 8 | MAGED1 |  |  | 258 | 0.545 | -0.1035 | No |
| 9 | NUDT4 |  |  | 318 | 0.502 | -0.1319 | No |
| 10 | GPC6 |  |  | 332 | 0.495 | -0.1363 | No |
| 11 | SORT1 |  |  | 577 | 0.406 | -0.2616 | No |
| 12 | CPEB2 |  |  | 588 | 0.401 | -0.2649 | No |
| 13 | TMSB4X |  |  | 590 | 0.401 | -0.2636 | No |
| 14 | NDUFB3 |  |  | 629 | 0.389 | -0.2815 | No |
| 15 | SEZ6L |  |  | 657 | 0.383 | -0.2938 | No |
| 16 | ID4 |  |  | 659 | 0.383 | -0.2925 | No |
| 17 | ADD3 |  |  | 674 | 0.378 | -0.2980 | No |
| 18 | NKAIN3 |  |  | 765 | 0.354 | -0.3433 | No |
| 19 | FSTL1 |  |  | 774 | 0.352 | -0.3458 | No |
| 20 | SLC38A2 |  |  | 785 | 0.350 | -0.3493 | No |
| 21 | TMTC4 |  |  | 795 | 0.348 | -0.3524 | No |
| 22 | LAPTM4A |  |  | 854 | 0.335 | -0.3810 | No |
| 23 | MDH1 |  |  | 924 | 0.322 | -0.4155 | No |
| 24 | ROBO2 |  |  | 963 | 0.316 | -0.4338 | No |
| 25 | SMIM14 |  |  | 985 | 0.312 | -0.4433 | No |
| 26 | MSI1 |  |  | 1051 | 0.300 | -0.4758 | No |
| 27 | CNR1 |  |  | 1180 | 0.282 | -0.5412 | No |
| 28 | CKB |  |  | 1200 | 0.279 | -0.5498 | No |
| 29 | JAM2 |  |  | 1204 | 0.279 | -0.5500 | No |
| 30 | PTPRE |  |  | 1243 | 0.275 | -0.5685 | No |
| 31 | ENC1 |  |  | 1287 | 0.270 | -0.5897 | No |
| 32 | ELAVL4 |  |  | 1358 | 0.261 | -0.6249 | No |
| 33 | GAPDH |  |  | 1361 | 0.261 | -0.6247 | No |
| 34 | KLHL9 |  |  | 1371 | 0.260 | -0.6282 | No |
| 35 | CSRP2 |  |  | 1386 | 0.258 | -0.6343 | No |
| 36 | SETD7 |  |  | 1458 | 0.251 | -0.6701 | No |
| 37 | RFC5 |  |  | 1473 | -0.250 | -0.6762 | No |
| 38 | MEX3A |  |  | 1474 | -0.251 | -0.6750 | No |
| 39 | DNA2 |  |  | 1479 | -0.252 | -0.6759 | No |
| 40 | DCLRE1C |  |  | 1493 | -0.257 | -0.6815 | No |
| 41 | ADRM1 |  |  | 1497 | -0.258 | -0.6818 | No |
| 42 | C2orf69 |  |  | 1500 | -0.259 | -0.6816 | No |
| 43 | HNRNPA2B1 |  |  | 1503 | -0.261 | -0.6815 | No |
| 44 | BLM |  |  | 1507 | -0.262 | -0.6818 | No |
| 45 | BRD8 |  |  | 1508 | -0.263 | -0.6805 | No |
| 46 | NIF3L1 |  |  | 1519 | -0.265 | -0.6845 | No |
| 47 | CEP78 |  |  | 1524 | -0.265 | -0.6853 | No |
| 48 | ZNF100 |  |  | 1536 | -0.268 | -0.6898 | No |
| 49 | ZNF43 |  |  | 1544 | -0.270 | -0.6922 | No |
| 50 | PSIP1 |  |  | 1564 | -0.275 | -0.7008 | No |
| 51 | KHDRBS1 |  |  | 1573 | -0.278 | -0.7036 | No |
| 52 | MRPL51 |  |  | 1576 | -0.279 | -0.7033 | No |
| 53 | FUBP1 |  |  | 1578 | -0.280 | -0.7025 | No |
| 54 | IQGAP3 |  |  | 1584 | -0.282 | -0.7038 | No |
| 55 | COX17 |  |  | 1589 | -0.284 | -0.7046 | No |
| 56 | LHX2 |  |  | 1597 | -0.285 | -0.7069 | No |
| 57 | RUVBL2 |  |  | 1599 | -0.286 | -0.7060 | No |
| 58 | RCC1 |  |  | 1604 | -0.286 | -0.7068 | No |
| 59 | CCDC150 |  |  | 1605 | -0.286 | -0.7054 | No |
| 60 | CNTRL |  |  | 1610 | -0.288 | -0.7061 | No |
| 61 | HP1BP3 |  |  | 1618 | -0.290 | -0.7084 | No |
| 62 | FAM83D |  |  | 1624 | -0.292 | -0.7096 | No |
| 63 | HES6 |  |  | 1632 | -0.294 | -0.7119 | No |
| 64 | RSRC1 |  |  | 1633 | -0.294 | -0.7105 | No |
| 65 | GAS2L3 |  |  | 1640 | -0.297 | -0.7122 | No |
| 66 | YAF2 |  |  | 1641 | -0.297 | -0.7108 | No |
| 67 | SUV39H2 |  |  | 1643 | -0.298 | -0.7099 | No |
| 68 | APOLD1 |  |  | 1648 | -0.299 | -0.7106 | No |
| 69 | NDUFAF3 |  |  | 1656 | -0.304 | -0.7128 | No |
| 70 | CEP128 |  |  | 1657 | -0.304 | -0.7114 | No |
| 71 | PARP2 |  |  | 1665 | -0.305 | -0.7136 | No |
| 72 | NUP107 |  |  | 1683 | -0.313 | -0.7210 | No |
| 73 | TMEM256 |  |  | 1685 | -0.314 | -0.7200 | No |
| 74 | G2E3 |  |  | 1687 | -0.315 | -0.7190 | No |
| 75 | NUDCD2 |  |  | 1691 | -0.316 | -0.7191 | No |
| 76 | ZNF93 |  |  | 1699 | -0.318 | -0.7213 | No |
| 77 | TACC1 |  |  | 1701 | -0.319 | -0.7203 | No |
| 78 | SNRPC |  |  | 1707 | -0.321 | -0.7214 | No |
| 79 | TRIM28 |  |  | 1709 | -0.322 | -0.7204 | No |
| 80 | CENPQ |  |  | 1711 | -0.322 | -0.7194 | No |
| 81 | PHLDA1 |  |  | 1718 | -0.324 | -0.7210 | No |
| 82 | SASS6 |  |  | 1719 | -0.325 | -0.7194 | No |
| 83 | CDC25C |  |  | 1724 | -0.327 | -0.7200 | No |
| 84 | VEZF1 |  |  | 1732 | -0.331 | -0.7220 | No |
| 85 | NEIL3 |  |  | 1733 | -0.331 | -0.7205 | No |
| 86 | PLK4 |  |  | 1740 | -0.336 | -0.7220 | No |
| 87 | STIL |  |  | 1744 | -0.337 | -0.7220 | No |
| 88 | EXOSC9 |  |  | 1746 | -0.338 | -0.7209 | No |
| 89 | TOP1 |  |  | 1749 | -0.340 | -0.7204 | No |
| 90 | CNIH4 |  |  | 1751 | -0.340 | -0.7193 | No |
| 91 | COA1 |  |  | 1753 | -0.341 | -0.7182 | No |
| 92 | SUZ12 |  |  | 1766 | -0.347 | -0.7228 | No |
| 93 | RAN |  |  | 1772 | -0.350 | -0.7237 | No |
| 94 | WDR76 |  |  | 1783 | -0.355 | -0.7273 | No |
| 95 | BRIP1 |  |  | 1790 | -0.357 | -0.7287 | Yes |
| 96 | KIF20A |  |  | 1792 | -0.358 | -0.7275 | Yes |
| 97 | POC1A |  |  | 1794 | -0.360 | -0.7264 | Yes |
| 98 | ILF2 |  |  | 1795 | -0.360 | -0.7247 | Yes |
| 99 | LGALS1 |  |  | 1799 | -0.362 | -0.7245 | Yes |
| 100 | ANAPC15 |  |  | 1802 | -0.362 | -0.7239 | Yes |
| 101 | TMEM158 |  |  | 1803 | -0.362 | -0.7221 | Yes |
| 102 | HNRNPC |  |  | 1804 | -0.362 | -0.7204 | Yes |
| 103 | CENPL |  |  | 1805 | -0.362 | -0.7187 | Yes |
| 104 | NEK2 |  |  | 1815 | -0.371 | -0.7216 | Yes |
| 105 | NUP50 |  |  | 1823 | -0.376 | -0.7235 | Yes |
| 106 | RHNO1 |  |  | 1824 | -0.377 | -0.7217 | Yes |
| 107 | XRCC6 |  |  | 1825 | -0.377 | -0.7200 | Yes |
| 108 | SAP30 |  |  | 1826 | -0.378 | -0.7182 | Yes |
| 109 | GJC1 |  |  | 1829 | -0.379 | -0.7174 | Yes |
| 110 | C18orf54 |  |  | 1830 | -0.380 | -0.7156 | Yes |
| 111 | LDHA |  |  | 1833 | -0.380 | -0.7149 | Yes |
| 112 | BARD1 |  |  | 1839 | -0.384 | -0.7157 | Yes |
| 113 | MASTL |  |  | 1843 | -0.385 | -0.7154 | Yes |
| 114 | MMS22L |  |  | 1846 | -0.387 | -0.7146 | Yes |
| 115 | SRSF10 |  |  | 1851 | -0.390 | -0.7149 | Yes |
| 116 | CEP135 |  |  | 1855 | -0.393 | -0.7146 | Yes |
| 117 | ACTL6A |  |  | 1859 | -0.393 | -0.7143 | Yes |
| 118 | KIF18B |  |  | 1860 | -0.393 | -0.7124 | Yes |
| 119 | FANCB |  |  | 1862 | -0.396 | -0.7111 | Yes |
| 120 | EXOSC8 |  |  | 1865 | -0.397 | -0.7102 | Yes |
| 121 | SMARCD1 |  |  | 1870 | -0.399 | -0.7104 | Yes |
| 122 | CHD7 |  |  | 1874 | -0.400 | -0.7101 | Yes |
| 123 | CBX1 |  |  | 1878 | -0.405 | -0.7098 | Yes |
| 124 | CENPO |  |  | 1884 | -0.410 | -0.7104 | Yes |
| 125 | RBMX |  |  | 1885 | -0.412 | -0.7085 | Yes |
| 126 | SHCBP1 |  |  | 1886 | -0.412 | -0.7065 | Yes |
| 127 | RFC4 |  |  | 1887 | -0.413 | -0.7046 | Yes |
| 128 | POLQ |  |  | 1891 | -0.417 | -0.7042 | Yes |
| 129 | YBX1 |  |  | 1892 | -0.417 | -0.7022 | Yes |
| 130 | SAE1 |  |  | 1893 | -0.417 | -0.7002 | Yes |
| 131 | RAD18 |  |  | 1898 | -0.419 | -0.7003 | Yes |
| 132 | DKC1 |  |  | 1904 | -0.428 | -0.7009 | Yes |
| 133 | E2F3 |  |  | 1907 | -0.429 | -0.6999 | Yes |
| 134 | HNRNPH3 |  |  | 1911 | -0.429 | -0.6995 | Yes |
| 135 | ANP32A |  |  | 1912 | -0.430 | -0.6974 | Yes |
| 136 | DCP2 |  |  | 1914 | -0.432 | -0.6959 | Yes |
| 137 | UHRF1 |  |  | 1921 | -0.438 | -0.6970 | Yes |
| 138 | DIAPH3 |  |  | 1924 | -0.439 | -0.6959 | Yes |
| 139 | LINC01224 |  |  | 1934 | -0.446 | -0.6985 | Yes |
| 140 | MZT1 |  |  | 1936 | -0.447 | -0.6969 | Yes |
| 141 | SNRPB |  |  | 1937 | -0.447 | -0.6948 | Yes |
| 142 | MZT2B |  |  | 1941 | -0.449 | -0.6942 | Yes |
| 143 | TTF2 |  |  | 1942 | -0.449 | -0.6921 | Yes |
| 144 | TPRKB |  |  | 1944 | -0.450 | -0.6905 | Yes |
| 145 | PSMA4 |  |  | 1946 | -0.451 | -0.6889 | Yes |
| 146 | CCNF |  |  | 1947 | -0.451 | -0.6868 | Yes |
| 147 | CLIC1 |  |  | 1949 | -0.454 | -0.6851 | Yes |
| 148 | NUDC |  |  | 1950 | -0.454 | -0.6830 | Yes |
| 149 | DDX39A |  |  | 1957 | -0.458 | -0.6840 | Yes |
| 150 | SEPHS1 |  |  | 1967 | -0.464 | -0.6865 | Yes |
| 151 | PTGES3 |  |  | 1970 | -0.467 | -0.6853 | Yes |
| 152 | CMC2 |  |  | 1977 | -0.473 | -0.6862 | Yes |
| 153 | CIT |  |  | 1978 | -0.474 | -0.6839 | Yes |
| 154 | HAUS6 |  |  | 1984 | -0.478 | -0.6843 | Yes |
| 155 | TIMELESS |  |  | 1989 | -0.482 | -0.6841 | Yes |
| 156 | PHIP |  |  | 1992 | -0.483 | -0.6829 | Yes |
| 157 | SKA1 |  |  | 1996 | -0.490 | -0.6821 | Yes |
| 158 | SPAG5 |  |  | 1997 | -0.490 | -0.6798 | Yes |
| 159 | PSMC3 |  |  | 1999 | -0.492 | -0.6780 | Yes |
| 160 | ARL6IP1 |  |  | 2001 | -0.494 | -0.6762 | Yes |
| 161 | FAM111A |  |  | 2002 | -0.497 | -0.6738 | Yes |
| 162 | TK1 |  |  | 2003 | -0.497 | -0.6715 | Yes |
| 163 | TUBB6 |  |  | 2006 | -0.500 | -0.6701 | Yes |
| 164 | HNRNPUL1 |  |  | 2009 | -0.501 | -0.6688 | Yes |
| 165 | CTCF |  |  | 2010 | -0.502 | -0.6664 | Yes |
| 166 | RANGAP1 |  |  | 2011 | -0.502 | -0.6641 | Yes |
| 167 | CDK5RAP2 |  |  | 2012 | -0.503 | -0.6617 | Yes |
| 168 | CHEK1 |  |  | 2013 | -0.504 | -0.6593 | Yes |
| 169 | MAGOHB |  |  | 2015 | -0.505 | -0.6574 | Yes |
| 170 | HIRIP3 |  |  | 2017 | -0.508 | -0.6556 | Yes |
| 171 | RRM1 |  |  | 2023 | -0.516 | -0.6557 | Yes |
| 172 | SRRT |  |  | 2025 | -0.517 | -0.6538 | Yes |
| 173 | HAUS8 |  |  | 2026 | -0.517 | -0.6514 | Yes |
| 174 | PARPBP |  |  | 2030 | -0.520 | -0.6505 | Yes |
| 175 | DEPDC1B |  |  | 2037 | -0.527 | -0.6511 | Yes |
| 176 | HELLS |  |  | 2038 | -0.528 | -0.6486 | Yes |
| 177 | CDCA3 |  |  | 2041 | -0.529 | -0.6471 | Yes |
| 178 | CCT5 |  |  | 2042 | -0.529 | -0.6446 | Yes |
| 179 | TROAP |  |  | 2046 | -0.533 | -0.6437 | Yes |
| 180 | TTK |  |  | 2047 | -0.533 | -0.6412 | Yes |
| 181 | HNRNPM |  |  | 2048 | -0.533 | -0.6386 | Yes |
| 182 | NCAPD3 |  |  | 2051 | -0.537 | -0.6371 | Yes |
| 183 | KIF22 |  |  | 2053 | -0.541 | -0.6351 | Yes |
| 184 | CENPJ |  |  | 2054 | -0.544 | -0.6325 | Yes |
| 185 | SAPCD2 |  |  | 2058 | -0.545 | -0.6315 | Yes |
| 186 | INCENP |  |  | 2061 | -0.556 | -0.6299 | Yes |
| 187 | STMN1 |  |  | 2063 | -0.567 | -0.6278 | Yes |
| 188 | SPDL1 |  |  | 2064 | -0.569 | -0.6251 | Yes |
| 189 | PTMA |  |  | 2065 | -0.570 | -0.6224 | Yes |
| 190 | DTL |  |  | 2066 | -0.573 | -0.6197 | Yes |
| 191 | FANCD2 |  |  | 2068 | -0.577 | -0.6175 | Yes |
| 192 | ZWINT |  |  | 2069 | -0.577 | -0.6147 | Yes |
| 193 | CEP152 |  |  | 2070 | -0.578 | -0.6120 | Yes |
| 194 | RTKN2 |  |  | 2071 | -0.579 | -0.6093 | Yes |
| 195 | RACGAP1 |  |  | 2072 | -0.579 | -0.6065 | Yes |
| 196 | CENPA |  |  | 2073 | -0.586 | -0.6038 | Yes |
| 197 | MND1 |  |  | 2074 | -0.586 | -0.6010 | Yes |
| 198 | LSM4 |  |  | 2076 | -0.587 | -0.5987 | Yes |
| 199 | SKA3 |  |  | 2077 | -0.590 | -0.5960 | Yes |
| 200 | BUB3 |  |  | 2078 | -0.596 | -0.5931 | Yes |
| 201 | AURKB |  |  | 2079 | -0.598 | -0.5903 | Yes |
| 202 | SRSF3 |  |  | 2081 | -0.606 | -0.5880 | Yes |
| 203 | CENPK |  |  | 2082 | -0.607 | -0.5851 | Yes |
| 204 | HNRNPR |  |  | 2083 | -0.612 | -0.5822 | Yes |
| 205 | USP1 |  |  | 2084 | -0.612 | -0.5793 | Yes |
| 206 | KNSTRN |  |  | 2085 | -0.615 | -0.5764 | Yes |
| 207 | CKAP5 |  |  | 2086 | -0.623 | -0.5734 | Yes |
| 208 | PLK1 |  |  | 2087 | -0.624 | -0.5705 | Yes |
| 209 | PIF1 |  |  | 2088 | -0.627 | -0.5675 | Yes |
| 210 | NCAPD2 |  |  | 2089 | -0.627 | -0.5646 | Yes |
| 211 | MCM8 |  |  | 2092 | -0.635 | -0.5626 | Yes |
| 212 | KIF18A |  |  | 2093 | -0.636 | -0.5596 | Yes |
| 213 | MIS18A |  |  | 2096 | -0.639 | -0.5576 | Yes |
| 214 | DNAJC9 |  |  | 2097 | -0.641 | -0.5546 | Yes |
| 215 | RFC3 |  |  | 2098 | -0.642 | -0.5515 | Yes |
| 216 | BRCA2 |  |  | 2100 | -0.650 | -0.5490 | Yes |
| 217 | PHF19 |  |  | 2101 | -0.650 | -0.5459 | Yes |
| 218 | ITGB3BP |  |  | 2102 | -0.652 | -0.5428 | Yes |
| 219 | GNG4 |  |  | 2103 | -0.652 | -0.5398 | Yes |
| 220 | NCAPH |  |  | 2104 | -0.652 | -0.5367 | Yes |
| 221 | CKAP2L |  |  | 2109 | -0.663 | -0.5356 | Yes |
| 222 | AURKA |  |  | 2110 | -0.673 | -0.5324 | Yes |
| 223 | CKS2 |  |  | 2115 | -0.694 | -0.5312 | Yes |
| 224 | CDC20 |  |  | 2117 | -0.695 | -0.5285 | Yes |
| 225 | RAD21 |  |  | 2118 | -0.697 | -0.5252 | Yes |
| 226 | ANP32B |  |  | 2119 | -0.700 | -0.5219 | Yes |
| 227 | CDCA2 |  |  | 2120 | -0.705 | -0.5185 | Yes |
| 228 | ZNF714 |  |  | 2121 | -0.705 | -0.5152 | Yes |
| 229 | PA2G4 |  |  | 2123 | -0.707 | -0.5124 | Yes |
| 230 | HDGF |  |  | 2124 | -0.709 | -0.5090 | Yes |
| 231 | CENPM |  |  | 2125 | -0.709 | -0.5057 | Yes |
| 232 | RAD51AP1 |  |  | 2127 | -0.715 | -0.5028 | Yes |
| 233 | CDCA8 |  |  | 2129 | -0.722 | -0.4999 | Yes |
| 234 | CKAP2 |  |  | 2131 | -0.729 | -0.4970 | Yes |
| 235 | ATAD5 |  |  | 2133 | -0.742 | -0.4940 | Yes |
| 236 | VRK1 |  |  | 2134 | -0.743 | -0.4905 | Yes |
| 237 | TUBA1C |  |  | 2135 | -0.756 | -0.4869 | Yes |
| 238 | KIF2C |  |  | 2136 | -0.756 | -0.4833 | Yes |
| 239 | KIF5B |  |  | 2137 | -0.756 | -0.4798 | Yes |
| 240 | BRCA1 |  |  | 2138 | -0.758 | -0.4762 | Yes |
| 241 | KIF14 |  |  | 2141 | -0.773 | -0.4736 | Yes |
| 242 | NDC80 |  |  | 2142 | -0.775 | -0.4699 | Yes |
| 243 | H2AFV |  |  | 2143 | -0.779 | -0.4662 | Yes |
| 244 | FBXO5 |  |  | 2144 | -0.779 | -0.4625 | Yes |
| 245 | KIF23 |  |  | 2145 | -0.782 | -0.4588 | Yes |
| 246 | GMNN |  |  | 2146 | -0.785 | -0.4551 | Yes |
| 247 | LMNB2 |  |  | 2147 | -0.786 | -0.4514 | Yes |
| 248 | MELK |  |  | 2148 | -0.786 | -0.4477 | Yes |
| 249 | ANLN |  |  | 2149 | -0.797 | -0.4439 | Yes |
| 250 | SMC3 |  |  | 2150 | -0.800 | -0.4401 | Yes |
| 251 | KIFC1 |  |  | 2151 | -0.802 | -0.4364 | Yes |
| 252 | DEPDC1 |  |  | 2152 | -0.809 | -0.4325 | Yes |
| 253 | DEK |  |  | 2154 | -0.817 | -0.4292 | Yes |
| 254 | NCAPG2 |  |  | 2156 | -0.827 | -0.4258 | Yes |
| 255 | TUBB |  |  | 2157 | -0.831 | -0.4219 | Yes |
| 256 | NUCKS1 |  |  | 2158 | -0.832 | -0.4179 | Yes |
| 257 | LSM5 |  |  | 2159 | -0.835 | -0.4140 | Yes |
| 258 | TUBB4B |  |  | 2162 | -0.843 | -0.4110 | Yes |
| 259 | ECT2 |  |  | 2163 | -0.846 | -0.4070 | Yes |
| 260 | HMGB3 |  |  | 2164 | -0.852 | -0.4030 | Yes |
| 261 | KIF20B |  |  | 2165 | -0.852 | -0.3990 | Yes |
| 262 | SPC24 |  |  | 2166 | -0.853 | -0.3950 | Yes |
| 263 | ARHGAP11A |  |  | 2167 | -0.855 | -0.3909 | Yes |
| 264 | DTYMK |  |  | 2168 | -0.858 | -0.3869 | Yes |
| 265 | MCM10 |  |  | 2169 | -0.858 | -0.3828 | Yes |
| 266 | BUB1 |  |  | 2170 | -0.860 | -0.3787 | Yes |
| 267 | FOXM1 |  |  | 2171 | -0.861 | -0.3747 | Yes |
| 268 | CDCA5 |  |  | 2172 | -0.866 | -0.3706 | Yes |
| 269 | LBR |  |  | 2173 | -0.870 | -0.3664 | Yes |
| 270 | BUB1B |  |  | 2174 | -0.871 | -0.3623 | Yes |
| 271 | NUF2 |  |  | 2176 | -0.877 | -0.3587 | Yes |
| 272 | CEP55 |  |  | 2177 | -0.883 | -0.3545 | Yes |
| 273 | KPNA2 |  |  | 2178 | -0.894 | -0.3503 | Yes |
| 274 | HMMR |  |  | 2179 | -0.895 | -0.3461 | Yes |
| 275 | CCDC34 |  |  | 2180 | -0.898 | -0.3418 | Yes |
| 276 | CCNA2 |  |  | 2181 | -0.902 | -0.3376 | Yes |
| 277 | CHAF1A |  |  | 2182 | -0.903 | -0.3333 | Yes |
| 278 | MIS18BP1 |  |  | 2183 | -0.903 | -0.3290 | Yes |
| 279 | SMS |  |  | 2184 | -0.910 | -0.3247 | Yes |
| 280 | KIF15 |  |  | 2185 | -0.915 | -0.3204 | Yes |
| 281 | KIF4A |  |  | 2186 | -0.921 | -0.3160 | Yes |
| 282 | PRR11 |  |  | 2187 | -0.930 | -0.3116 | Yes |
| 283 | ORC6 |  |  | 2188 | -0.937 | -0.3072 | Yes |
| 284 | C21orf58 |  |  | 2189 | -0.937 | -0.3028 | Yes |
| 285 | CENPW |  |  | 2192 | -0.943 | -0.2994 | Yes |
| 286 | TUBA1B |  |  | 2193 | -0.947 | -0.2949 | Yes |
| 287 | LMNB1 |  |  | 2194 | -0.950 | -0.2904 | Yes |
| 288 | HJURP |  |  | 2195 | -0.962 | -0.2858 | Yes |
| 289 | UBE2T |  |  | 2196 | -0.965 | -0.2813 | Yes |
| 290 | FANCI |  |  | 2198 | -0.966 | -0.2772 | Yes |
| 291 | CDKN3 |  |  | 2199 | -0.979 | -0.2726 | Yes |
| 292 | CCNB2 |  |  | 2200 | -0.983 | -0.2680 | Yes |
| 293 | CKS1B |  |  | 2201 | -0.987 | -0.2633 | Yes |
| 294 | ESCO2 |  |  | 2204 | -1.004 | -0.2596 | Yes |
| 295 | SMC2 |  |  | 2205 | -1.006 | -0.2548 | Yes |
| 296 | KIF11 |  |  | 2206 | -1.008 | -0.2501 | Yes |
| 297 | EZH2 |  |  | 2207 | -1.017 | -0.2452 | Yes |
| 298 | SPC25 |  |  | 2208 | -1.019 | -0.2404 | Yes |
| 299 | XRCC2 |  |  | 2210 | -1.028 | -0.2361 | Yes |
| 300 | CENPE |  |  | 2211 | -1.036 | -0.2312 | Yes |
| 301 | NUDT1 |  |  | 2214 | -1.054 | -0.2272 | Yes |
| 302 | CLSPN |  |  | 2216 | -1.060 | -0.2228 | Yes |
| 303 | CENPN |  |  | 2217 | -1.079 | -0.2177 | Yes |
| 304 | UBE2C |  |  | 2218 | -1.081 | -0.2125 | Yes |
| 305 | CENPU |  |  | 2219 | -1.088 | -0.2074 | Yes |
| 306 | H2AFZ |  |  | 2220 | -1.090 | -0.2022 | Yes |
| 307 | HMGB1 |  |  | 2221 | -1.116 | -0.1970 | Yes |
| 308 | NCAPG |  |  | 2222 | -1.122 | -0.1917 | Yes |
| 309 | H2AFX |  |  | 2223 | -1.146 | -0.1862 | Yes |
| 310 | CCNB1 |  |  | 2224 | -1.148 | -0.1808 | Yes |
| 311 | CDK1 |  |  | 2225 | -1.153 | -0.1754 | Yes |
| 312 | PTTG1 |  |  | 2226 | -1.198 | -0.1697 | Yes |
| 313 | ANP32E |  |  | 2227 | -1.200 | -0.1640 | Yes |
| 314 | MAD2L1 |  |  | 2228 | -1.204 | -0.1583 | Yes |
| 315 | GTSE1 |  |  | 2230 | -1.220 | -0.1531 | Yes |
| 316 | DBF4 |  |  | 2232 | -1.247 | -0.1477 | Yes |
| 317 | HMGN2 |  |  | 2233 | -1.267 | -0.1417 | Yes |
| 318 | RRM2 |  |  | 2234 | -1.268 | -0.1357 | Yes |
| 319 | DLGAP5 |  |  | 2235 | -1.273 | -0.1297 | Yes |
| 320 | SMC4 |  |  | 2236 | -1.281 | -0.1236 | Yes |
| 321 | PBK |  |  | 2237 | -1.325 | -0.1174 | Yes |
| 322 | ATAD2 |  |  | 2238 | -1.381 | -0.1108 | Yes |
| 323 | TMPO |  |  | 2239 | -1.382 | -0.1043 | Yes |
| 324 | PRC1 |  |  | 2240 | -1.397 | -0.0977 | Yes |
| 325 | HMGB2 |  |  | 2241 | -1.469 | -0.0907 | Yes |
| 326 | BIRC5 |  |  | 2242 | -1.469 | -0.0838 | Yes |
| 327 | HIST1H1C |  |  | 2243 | -1.485 | -0.0768 | Yes |
| 328 | ASPM |  |  | 2244 | -1.490 | -0.0697 | Yes |
| 329 | TOP2A |  |  | 2246 | -1.562 | -0.0629 | Yes |
| 330 | NUSAP1 |  |  | 2247 | -1.600 | -0.0553 | Yes |
| 331 | TPX2 |  |  | 2248 | -1.609 | -0.0477 | Yes |
| 332 | CENPF |  |  | 2249 | -1.691 | -0.0397 | Yes |
| 333 | HIST1H1D |  |  | 2250 | -1.720 | -0.0316 | Yes |
| 334 | HIST1H1B |  |  | 2251 | -1.796 | -0.0231 | Yes |
| 335 | MKI67 |  |  | 2252 | -1.892 | -0.0141 | Yes |
| 336 | HIST1H4C |  |  | 2253 | -2.984 | 0.0000 | Yes |
Table: GSEA details [plain text format]

  

Fig 2: RICHARDS\_NOWAKOWSKI\_SCIENCE\_2017\_IPC\_DIV1\_UPREG: Random ES distribution      
 Gene set null distribution of ES for **RICHARDS\_NOWAKOWSKI\_SCIENCE\_2017\_IPC\_DIV1\_UPREG**
